# Supplementary material for: Comparison of Ki-67 Labeling Index Patterns of Diffuse Large B-Cell Lymphomas and Burkitt Lymphomas Using Image Analysis: A Multicenter Study
Source: Diagnostics (Basel). 2021 Feb 19;11(2):343. doi: 10.3390/diagnostics11020343 (PMC7922648; doi:10.3390/diagnostics11020343)
Supplement: Supplementary file 1 [file diagnostics-11-00343-s001.pdf]

diagnostics MDPI

Supplementary Table S1. Comparison of Ki-67 labeling index patterns between Burkitt lymphomas (BLs), activated B-cell (ABC), and germinal center cell (GCB) subtype diffuse large B-cell lymphomas (DLBCLs).

		BLs (n = 29)	DLBCL, ABC type (n = 73)	DLBCL, GCB type (n = 30)	p-value
**AGE**	years	51.0 ± 24.8	62.8 ± 14.5	60.5 ± 15.2	0.043
SEX					
Male	No.	18 (62.1%)	39 (53.4%)	22 (73.3%)	0.167
Female	No.	11 (37.9%)	34 (46.6%)	8 (26.7%)	
M:F		1:0.61	1:0.87	1:0.36	
Total counted cells	No.	5752.6 ± 786.2	5587.5 ± 541.1	5753.2 ± 757.6	0.983
Negative cells	No.	337.4 ± 385.4	656.5 ± 767.6	772.6 ± 571.4	***0.013***
	%	5.9 ± 6.6	11.7 ± 14.0	13.9 ± 11.1	***0.013***
Positive cells	No.	5415.1 ± 850.9	4931.0 ± 905.0	4980.6 ± 997.1	0.076
	%	94.1 ± 6.6	88.3 ± 14.0	86.1 ± 11.1	***0.013***
1+	No.	37.8 ± 24.9	871.7 ± 599.3	595.8 ± 577.8	***0.001***
	%	0.7 ± 0.5	15.6 ± 10.8	10.8 ± 10.8	***0.001***
2+	No.	163.8 ± 102.3	1146.3 ± 570.4	718.6 ± 333.6	***< 0.001***
	%	2.9 ± 1.7	20.4 ± 9.9	12.4 ± 5.4	***0.001***
3+	No.	5213.6 ± 825.7	2913.0 ± 1256.9	3666.2 ± 1227.8	***< 0.001***
	%	90.6 ± 6.2	52.2 ± 22.2	62.9 ± 16.8	***< 0.001***
Proportion of 1+ in positive cells	%	0.7 ± 0.5	18.1 ± 12.6	12.9 ± 12.8	***< 0.001***
Proportion of 2+ in positive cells	%	3.0 ± 1.8	24.2 ± 13.1	14.6 ± 5.9	***0.001***
Proportion of 3+ in positive cells	%	96.3 ± 2.0	57.7 ± 20.8	72.6 ± 15.4	***< 0.001***
Proportion of 1+/2+ in positive cells	%	3.7 ± 2.0	42.3 ± 20.8	27.4 ± 15.4	***< 0.001***
Proportion of 2+/3+ in positive cells	%	99.3 ± 0.5	81.9 ± 12.6	87.2 ± 12.3	***< 0.001***

Significant p-values are bolded and italicized.

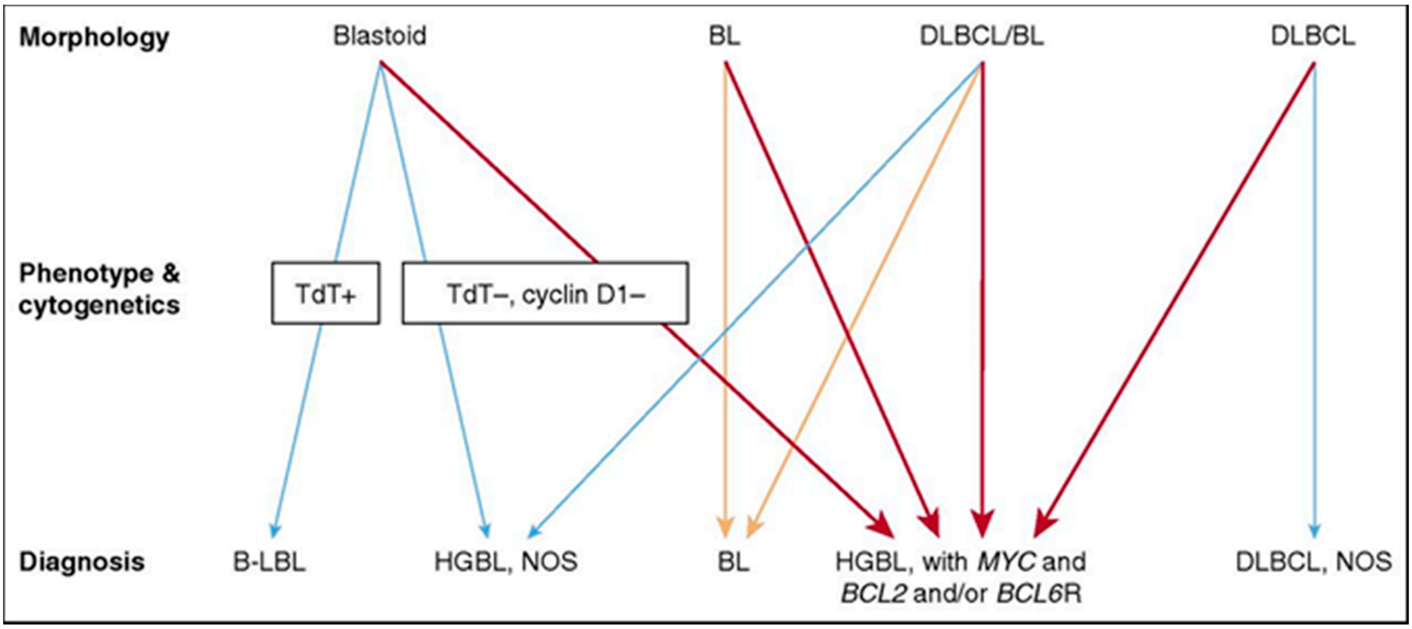

Supplementary Figure S1. Classification algorithm for high grade B-cell lymphomas (HGBLs) in the 2018 World Health Organization Classification.
